# Phylometagenomics of cycad coralloid roots reveals shared symbiotic signals

**DOI:** 10.1099/mgen.0.001207

**Published:** 2024-03-07

**Authors:** Edder D. Bustos-Diaz, Arely Cruz-Perez, Diego Garfias-Gallegos, Paul M. D'Agostino, Michelle M. Gehringer, Angelica Cibrian-Jaramillo, Francisco Barona-Gomez

**Affiliations:** 1 Evolution of Metabolic Diversity Laboratory, Unidad de Genómica Avanzada (Langebio), Cinvestav, Irapuato, Guanajuato, Mexico; 2 Institute of Biology, Leiden University, Netherlands, 2333 BE, Leiden; 3 Ecological and Evolutionary Genomics Laboratory, Unidad de Genómica Avanzada (Langebio), Cinvestav, Irapuato, Guanajuato, Mexico; 4 Chair of Technical Biochemistry, Technical University of Dresden, Bergstraße 66, 01069 Dresden, Germany; 5 Department of Microbiology, University of Kaiserslautern-Landau (RPTU), 67663 Kaiserslautern, Germany; 6 Naturalis Biodiversity Center, Leiden 2333 CR, Netherlands

**Keywords:** *Aulosira*, cyanobiont, cycad coralloid roots, *Nostocales*, phylometagenomics, symbiosis

## Abstract

Cycads are known to host symbiotic cyanobacteria, including *Nostocales* species, as well as other sympatric bacterial taxa within their specialized coralloid roots. Yet, it is unknown if these bacteria share a phylogenetic origin and/or common genomic functions that allow them to engage in facultative symbiosis with cycad roots. To address this, we obtained metagenomic sequences from 39 coralloid roots sampled from diverse cycad species and origins in Australia and Mexico. Culture-independent shotgun metagenomic sequencing was used to validate sub-community co-cultures as an efficient approach for functional and taxonomic analysis. Our metanalysis shows a host-independent microbiome core consisting of seven bacterial orders with high species diversity within the identified taxa. Moreover, we recovered 43 cyanobacterial metagenome-assembled genomes, and in addition to *Nostoc* spp., symbiotic cyanobacteria of the genus *Aulosira* were identified for the first time. Using this robust dataset, we used phylometagenomic analysis to reveal three monophyletic cyanobiont clades, two host-generalist and one cycad-specific that includes *Aulosira* spp. Although the symbiotic clades have independently arisen, they are enriched in certain functional genes, such as those related to secondary metabolism. Furthermore, the taxonomic composition of associated sympatric bacterial taxa remained constant. Our research quadruples the number of cycad cyanobiont genomes and provides a robust framework to decipher cyanobacterial symbioses, with the potential of improving our understanding of symbiotic communities. This study lays a solid foundation to harness cyanobionts for agriculture and bioprospection, and assist in conservation of critically endangered cycads.

## Data Summary

Metagenomic sequencing raw data and metagenome assembled genomes (MAGs) are available under NCBI bioproject PRJNA1014740 and JGI Seq Project IDs 1 325 811, 1 325 813, 1 325 814, 1 325 815 and 1 325 816. Specific biosample and seq project IDs per metagenome and MAG are given in Tables 1 and 2, respectively.

**Impact Statement**

Symbiotic *Nostocales,* or cyanobionts, and their associated bacterial communities can interact with many hosts, including cycads, one group of highly endangered gymnosperms, but it is unclear if they are opportunistic or have evolved to adapt to their hosts. Here, we showed that there are seven bacterial orders, including the *Nostocales* cyanobionts, commonly found in the bacterial communities. These cyanobionts, from cycads and other hosts, are phylogenetically related and mostly concentrated in three symbiotic clades. One of these clades was found to be exclusively composed of cyanobionts from Mexican cycads and included members of the genus *Aulosira*, suggesting specialization and possible convergent evolution of symbiosis.

## Introduction

Cycads are a group of gymnosperms whose origin has been traced to the Carboniferous [1] and are currently endangered due to poaching and habitat loss [2]. These plants can harbour symbiotic *Nostocales,* known as cyanobionts, inside specialized coralloid roots, a unique symbiotic organ among gymnosperms [3,4]. These cyanobionts are facultative, recruited from the soil for a transient symbiosis. Recently, it has become apparent that coralloid roots also contain other sympatric bacteria, such as *Hypomicrobiales* and *Caulobacterales* [5,8]. Biological nitrogen fixation (BNF) is believed to be the main function of the coralloid root microbiome, in exchange for carbon sources from the host [9,10]. Indeed, in nitrogen-poor environments, cycad leaves carry the same nitrogen fractionation signal as their diazotrophic cyanobionts, confirming the plant’s reliance on symbiotic BNF [11]. Other less studied functions for cycad cyanobionts and associated microbes might also be relevant for the symbiosis, such as diverse biological and ecological functions supported by natural products or specialized metabolites [12,14].

All known cycad species can develop coralloid roots, but not all *Nostocales* species are recruited for symbiosis [15]. Most characterized cycad cyanobionts belong to the family *Nostocaceae*, with *Calothrix* species being isolated on rare occasions [16,18]. Previous morphological [4] and single-gene studies targetting the 16S rRNA gene or the tRNA-Leu intron [16,19] show that cycad cyanobionts are phylogenetically related to the facultative cyanobionts from *Gunnera* [20], bryophytes [12,21] and lichens [22,23]. Whole-genome analyses partially support this pattern, although these studies are limited as they vary in their sampling and the number and composition of presumed symbiotic clades [5,21, 24,27].

It has been hypothesized that cyanobionts isolated from different hosts with close phylogenetic relationships might have shared symbiosis genes (i.e. genes unique to and conserved in cyanobionts used to form and/or maintain the symbiosis) [28]. Even though some genes have been experimentally shown to be needed for the symbiosis, such as the hormogonia regulation locus *hrmUA* [29], or the genes *ntcA, hetR* and *hetF*, which are required for heterocyst development [30], these are commonly found throughout *Nostocales*. Thus, the specific genes related to symbiosis remain unknown. Since many cyanobionts show the ability to form symbiotic relationships with different hosts, showing a ‘symbiotic plastic’ behaviour [15,25, 31,33], the possibility of a symbiotic genomic signature within the context of symbiotic plasticity is also an interesting hypothesis. However, the incomplete phylogenomic framework to date limits comparative analyses aimed to identify common genomic functions related to the mechanisms that underlie cycad and other hosts’ cyanobiont mutualisms. Not surprisingly, the search for symbiotic genes have yielded incongruent results [25,27]. Warshan and collaborators found a group of 74 genes that were present in the sequenced cyanobiont, but not in the free-living strains. However, a later study on a larger genomic dataset found [27] that free-living strains also contain these genes. Free-living strains are those found outside of a symbiotic host, although this term does not necessarily imply that the strain is incapable of forming symbiotic relationships. Therefore, it is currently unknown if the phylogenetic relationship among cyanobionts is congruent with specific symbiosis genes or broader symbiotic genomic signatures, such as those found in the diazotrophic symbiosis between *Rhizobiales* and legumes (e.g. *nod* genes) [34].

Just as the putative shared symbiotic genes remain to be identified, it is also unknown if the non-cyanobacterial sympatric bacterial communities of the coralloid root have a role in the symbiotic behaviour of cyanobionts. These communities have been independently identified in lichens, bryophytes and *Azolla* [35,38], in addition to coralloid roots [5,8]. However, even when these microbiomes seem similar, no formal meta-analysis of these datasets has been done, which would be an important first step to test their overall roles during symbiosis, including symbiotic plasticity. The latter is an interesting possibility, as cyanobacteria-associated communities have been shown to interact with the cyanobiont in coralloid roots [5] and to directly contribute to the *Azolla* symbiosis by complementary denitrification [37].

The present study aims to describe cycad-associated microbiomes and the phylogenetic placement of the cyanobiont, in order to identify symbiotic genes or genomic signatures in cyanobacteria associated with cycads and beyond. We began with a taxonomic assessment of the symbiotic communities of coralloid roots and its meta-analysis, coupled with a phylometagenomic reconstruction of the newly generated metagenome assembled genomes (MAGs) of sympatric cyanobionts. These MAGs were added to existing high-quality genomes from cyanobionts and free-living strains (i.e. those obtained from *Nostocales* isolated outside a symbiotic host) in order to find well-supported phylogenetically related groups of cyanobionts. The genomes in the identified symbiotic clades, in turn, were used for pangenomic comparisons to identify conserved genes specific to the symbiotic genomes. The generated data, which quadruple the number of publicly available cycad cyanobiont genomes, clarifies the phylogenomic distribution of cyanobionts from cycads and other systems and confirms the existence of at least three symbiotic lineages with a relevant functional and phylogenetic signal, or genomic signature, that warrants targeted experimental characterization.

## Methods

### Sample collection and co-culture inoculation

Coralloid roots were collected from different geographical locations with the appropriate permissions, as described in Table 1. All coralloid roots were transported to the laboratory and washed before endophyte extraction. The washing was done by cleaning the root surface with tap water; they were then submerged in sterilized milliQ water and vortexed for 3 min. After the initial wash, four more washing steps were done, with hydrogen peroxide, sterilized milliQ water, a chlorine solution at 6 % concentration and sterilized milliQ water again for 3, 1, 3 and 1 min, respectively. After the last step, 200 µl of the water was used to inoculate a plate with BG-11_0_ [5] to check that no rhizospheric micro-organisms remained. Then, the tip of the coralloid ‘fingers’ was cut with a sterile scalpel and the cyanobacterial zone biomass was collected with a sterile syringe tip. Biomass used for co-cultures was inoculated in BG-11_0_ and grown in a light/dark cycle of 16/8 h at 22 °C, until they were visually saturated. Biomass used for direct extractions was obtained by washing the roots as above, and then extracting as much biomass as possible from within the coralloid root ‘fingers’. Once collected, the biomass was stored at −20 °C prior to DNA extraction.

**Table 1.** Sampling information

Sample code name	Cycad	Location	Environment	Extraction method	Biosample	DSM no.	Reference
ChiQUE01	*Ceratozamia hildae*	Queretaro, Mexico	WP	EC	SAMN37340527*	na	This study
ChiQUE02	*Ceratozamia hildae*	Queretaro, Mexico	WP	DE	SAMN37340528*	na	This study
ChiSLP01	*Ceratozamia hildae*	San Luis Potosi, Mexico	WP	EC	SAMN37340529*	na	This study
ChiSLP02	*Ceratozamia hildae*	San Luis Potosi, Mexico	WP	EC	SAMN37340530*	na	This study
ChiSLP03	*Ceratozamia hildae*	San Luis Potosi, Mexico	WP	DE	SAMN37340531*	na	This study
ChiVER01	*Ceratozamia hildae*	Veracruz, Mexico	BG	EC	SAMN37340532*	na	This study
CmiSLP01	*Ceratozamia microstrobila*	San Luis Potosi, Mexico	WP	EC	SAMN37340533*	na	This study
CmiVER01	*Ceratozamia microstrobila*	Veracruz, Mexico	BG	EC	SAMN37340534*	na	This study
CreGUA01	*Cycas revoluta*	Guanajuato, Mexico	BG	EC	SAMN37340535*	na	This study
DcaGUA01	*Dioon caputoi*	Guanajuato, Mexico	BG	EC	SAMN37340536*	na	This study
DedQUE01	*Dioon edule*	Queretaro, Mexico	WP	EC	SAMN37340537*	na	This study
DedQUE02	*Dioon edule*	Queretaro, Mexico	WP	EC	SAMN37340538*	na	This study
DedQUE03	*Dioon edule*	Queretaro, Mexico	WP	EC	SAMN37340539*	na	This study
DedQUE04	*Dioon edule*	Queretaro, Mexico	WP	EC	SAMN37340540*	na	This study
DedQUE05	*Dioon edule*	Queretaro, Mexico	WP	EC	SAMN37340541*	na	This study
DedQUE07	*Dioon edule*	Queretaro, Mexico	WP	EC	SAMN37340542*	na	This study
DedQUE08	*Dioon edule*	Queretaro, Mexico	WP	EC	SAMN37340543*	na	This study
DedQUE09	*Dioon edule*	Queretaro, Mexico	WP	DE	SAMN37340544*	na	This study
DedQUE10	*Dioon edule*	Queretaro, Mexico	WP	DE	SAMN37340545*	na	This study
DedQUE11	*Dioon edule*	Queretaro, Mexico	WP	DE	SAMN37340546*	na	This study
DedQUE12	*Dioon edule*	Queretaro, Mexico	WP	DE	SAMN37340547*	na	This study
DedSLP01	*Dioon edule*	San Luis Potosi, Mexico	WP	EC	SAMN37340548*	na	This study
DedSLP03	*Dioon edule*	San Luis Potosi, Mexico	WP	DE	SAMN37340549*	na	This study
DedSLP04	*Dioon edule*	San Luis Potosi, Mexico	WP	DE	SAMN37340550*	na	This study
DedSLP05	*Dioon edule*	San Luis Potosi, Mexico	WP	DE	SAMN37340551*	na	This study
DedVER01	*Dioon edule*	Veracruz, Mexico	BG	EC	SAMN37340552*	na	This study
DedVER02	*Dioon edule*	Veracruz, Mexico	BG	EC	SAMN37340553*	na	This study
EfeVER01	*Encephalartos ferox*	Veracruz, Mexico	BG	EC	SAMN37340554*	na	This study
EkiNYC01	*Encephalartos kisambo*	New York City, USA	BG	EC	SAMN37340555*	na	This study
EspVER01	*Encephalartos* sp*.*	Veracruz, Mexico	BG	EC	SAMN37340556*	na	This study
SerVER01	*Stangeria eriopus*	Veracruz, Mexico	BG	EC	SAMN37340557*	na	This study
ZfuCHP01	*Zamia furfuracea*	Chiapas, Mexico	WP	EC	SAMN37340558*	na	This study
ZfuVER01	*Zamia furfuracea*	Veracruz, Mexico	BG	EC	SAMN37340559*	na	This study
ZfuVER08	*Zamia furfuracea*	Veracruz, Mexico	WP	EC	SAMN37340560*	na	This study
Nostoc 1.3	*Bowenia serrulata*	Byfield, Australia	WP	EC	1 325 816†	DSM114160	[17]
Nostoc 40.5	*Macrozamia communis*	Currambene, Australia	WP	EC	1 325 815†	DSM114169	[17]
Nostoc 62.1	*Macrozamia mountperriensis*	Brooweena, Australia	WP	EC	1 325 813†	DSM114159	[17]
Nostoc 73.1	*Macrozamia serpentina*	Mt. Slopeway, Australia	WP	EC	1 325 811†	DSM114167	[17]
Nostoc 74.5	*Macrozamia macleayi*	Mt. Colosseum, Australia	WP	EC	1 325 814†	DSM114161	[17]

a *NCBI biosample number.

b †JGI Seq Project ID.

BG Botanical garden DE Direct extraction EC Extraction from co-culture WP Wild populations

### DNA extraction and sequencing

Biomass for DNA extraction was obtained from one visually saturated co-culture plate collected using a sterile loop, or from obtained directly from within the coralloid root after these were surface sterilized. Biomass was then flash-frozen with liquid nitrogen and grround in a mortar. This ground material was then resuspended in 1 ml of TE buffer (Tris 30 mM, EDTA 10 mM, SDS 1 %; pH 8), and 10 µl of RNAse (10 mg ml^−1^), 10 µl of proteinase K (20 mg ml^−1^) and 0.05 g of lysozyme from chicken egg white (Sigma) were added. The solution was incubated at 37 °C for 30 min, after which 400 µl of NaCl (5 M) and 300 µl of CTAB/NaCl were added. The resoluting solution was incubated again at 65 °C for 20 min and extraction was done with chloroform–isoamyl alcohol (24 : 1). DNA was then precipitated with isopropanol and resuspended in sterilized MilliQ water. DNA quality was checked by NanoDrop.

All samples from American cycads, from co-cultures and direct extractions, were sequenced through shotgun sequencing at Novogene (USA) with Illumina HiSeq 2×150 paired-end (PE), or at Labsergen-langebio (Mexico) with Illumina MiSeq (2×150 PE), Illumina NextSeq (2×150 PE), Illumina NovaSeq (2×150 PE) or MGI tech MGISEQ-2000 (2×150 PE) with the exception of sample ZfuVER08 which was sequenced using Illumina MiSeq (2×100 PE) at Labsergen-langebio (Mexico). For cyanobacteria derived from Australian cycads, sequencing was performed at the Joint Genome Institute (JGI) using the PacBio Sequel II system. The JGI platform performed sequencing, assembly annotation and automatic binning of genomes. The quality of all reads obtained through short read shotgun sequencing (Illumina and MGISEQ) was visually checked with MultiQC [39].0. Additionally, all trimmed reads were first mapped against the *Homo sapiens* GRCh38 no-alt analysis set provided in the Bowtie2 webpage using Bowtie2 v10.4.0 in local mode [40] to remove all reads from human origin. Remaining reads were corrected with the BayesHammer module of SPAdes v3.15.2 [41].

### Taxonomic analysis of bacterial communities

The resulting forward and reverse reads obtained from Illumina and MGISEQ short read sequencing, as well as filtered circular consensus reads (ccs) from Pacbio sequencing, were used for metagenomic assembly and taxonomic classification using Kraken2 version 2.1.2 [42] with the Standard database (release 14 March 2023) from https://benlangmead.github.io/aws-indexes/k2. Kraken2 output files were exported to biom using the kraken-biom script [43] and imported into R for downstream analysis using the phyloseq [44] and ampvis2 [45] libraries. All eukaryotic operational taxonomic units (OTUs) and bacterial OTUs with fewer than 100 reads were removed. Rarefaction of metagenomes from Queretero and San Luis Potosi populations was done using mirlyn [46]. Each metagenome was rarefied ten times and observed, Shannon and inverse Simpson diversity indexes were calculated for each repetition using phyloseq [44]. Obtained values were gathered, and the average of each metric per metagenome was used for statistical testing of differences between extraction methodologies and graph generation. The statistical significance of changes in alpha diversity due to extraction methodology was calculated using the Wilcoxon sum rank test. To obtain OTUs shared between extraction methodologies from both populations, the number of shared and unique OTUs per rarefied community were calculated using ampvis2 [45] and the average was used for the final figure. All non-rarefied metagenomes were used to generate the core microbiome. The core was calculated using the microbiome R package [47] and consisted of all bacterial OTUs at the order level present in at least 80 % of the samples. After obtaining the core, all research articles mentioned in Table S2 (available in the online version of this article) were checked to retrieve all bacterial orders found in the bacterial communities associated with the cyanobionts. Naming conventions were homogenized before Venn diagram generation.

### Metagenomic assembly and binning to obtain MAGs

For the assembly step, two programs were used, metaSPAdes version 3.15.5 [48] and Megahit version 1.2.9 [49]. The resulting assemblies were then used to obtain bins with MaxBin version 2.2.7 [50] and metaBAT version 2.14 [51]. After metagenomic assembly and binning, DAS TOOL version 1.1.5 was used to obtain the best quality MAGs per metagenome [52] and GTBD-TK [53] was used to classify them. All MAGs that belonged to *Nostocales* were selected and checked with CheckM2 version 1.0.2 [54] to ensure that all had high completeness (>95 %) and low contamination (<5 %) values. For the long-read shotgun metagenomes, the JGI metagenomic and binning pipeline was used. *Nostocales* MAGs recovered in this way were also classified and checked using the same methodology used for all other MAGs (Table S3). All the recovered MAGs were checked for completeness using the nostocales_db10 dataset of the single-copy gene markers BUSCO database (BUSCOs) which contained 1899 genes [55].

### Phylogenomic and functional analysis

328 *Nostocales* genomes were recovered from the NCBI GenBank and JGI Gold databases for phylogenetic and pangenome analysis. The criteria used to choose genomes was that these must have fewer than 1200 contigs, more than 90 % complete BUSCOs from the nostocales_odb10 dataset [55] and more than 85 % of contigs taxonomically assigned to *Nostocales* using kraken2 with the same genome database used for the taxonomic analysis [42] (Table S4). After quality control, all genomes from the databases, plus all the recovered *Nostocales* MAGs and the genome of *Chlorogloea* sp*.* CCALA 695 were annotated with Bakta version 1.7.0 [56] to ensure homogeneity. After this, an approximate maximum likelihood phylogenomic tree was reconstructed using the GToTree pipeline version 1.8.2 using the 251 Cyanobacteria-specific single copy gene set from the same program [57]. The resulting tree was annotated using iTOL [58] and rooted with *Chlorogloea* sp*.* CCALA 695. The super-clade that contained most cycanobionts was singled out from the general phylogeny for further analysis. Clades within the super-clade were defined by average nucleotide identity (ANI) similarity (Fig. S3). The three identified clades composed of more than 50 % of cyanobiont genomes were called SYMB-1, 2 and 3. The remaining clades, mostly composed of free-living species, were named FL-I to XI.

All the 209 genomes from the super-clade were used to generate a pangenome using PPanGGOLiN version 1.2.105 [59] (Table S5). Posterior analysis and figure generation were carried out in R. The pagoo library [60] was used to generate pangenome curves and the micropan library [61] was used to calculate Heap’s law alpha values. To look for ‘symbiosis genes’, all gene families from the shell and cloud partition present in each of the symbiotic clades were recovered. All genomes in each clade were grouped as symbiotic and free-living according to the metadata obtained from each genome. Cyanobionts from cycads were separated from the symbiotic group to look for cycad-specific symbiosis genes. Prevalence, as a percentage of genomes in each group that contained a specific gene family, was calculated for all the selected gene families of each group. All the genomes from the FL clades were grouped and incorporated in the analysis except for the analysis of SYMB-1. For this, FL genomes were separated into more groups, to consider closely related genomes. Only those whose prevalence was >60 % were coloured green.

For functional analysis, all the annotated genomes from the 209 *Nostocales* in the super-clade were searched against the COGs database using COGclassifier version 1.0.5 [62] (Table S6). After this, the percentual fraction of genes classified in each COG per genome was calculated. The Wilcoxon sum rank test was used to compare the percentage of COG genes in each category for all genomes in each SYMB clade against all FL genomes, and those with a *P* value <0.001 were considered significant. To find differential COGs, the genes that were only present in at least one genome in one or more symbiotic clade were recovered from the pangenome. COG annotation files of all genomes from the SYMB clades were checked to detect how many of the COGs in each category were differential. The ratio between the number of differential COGs and total COGs per genome was calculated for all these genomes and presented graphically by grouping by SYMB clade.

## Results

### Taxonomic characterization of cycad metagenomes revealed a common core

**Fig. 1.**
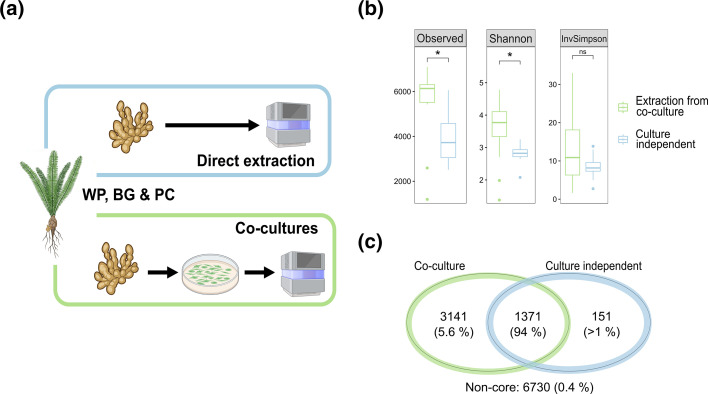
Direct culture-independent extraction and co-culture strategies to study cycad coralloid root symbiotic communities. (**a**) Two methodologies were used for metagenomic sequencing, with (co-cultures) or without (direct extraction) co-culturing in BG-11_0_ prior to DNA extraction. Diagram created with BioRender.com. (**b**) Overall taxonomic diversity between samples from each methodology was similar, but abundance was changed. (**c**) Despite changes in abundance, dominant taxa remained the same.

To capture the microbial taxonomic diversity of cycad coralloid roots, we adopted a combined approach including co-cultures [5], from both botanical gardens (BG) or private collections (PC), and wild populations (WP) from Mexico and Australia [17]; and culture-independent shotgun metagenomics of WP specimens from Mexican cycads (Fig. 1a). In total, 39 microbial communities from 15 different cycad species of the genera *Bowenia, Ceratozamia, Cycas, Dioon, Encephalartos, Macrozamia, Stangeria* and *Zamia* were obtained (Table 1). Of these, nine corresponded to BG, two to PC and 28 to WP samples. The DNA of all samples was extracted and used for metagenomic sequencing, taxonomic read assignment and metagenome assembly. Moreover, to investigate the effect of co-culturing on the composition of symbiotic communities, whole communities from two WP (Queretaro and San Luis Potosi, Mexico) were sequenced from both direct extractions and co-cultures, allowing for a direct comparison.

Firstly, the Alpha diversity values for all co-culture and culture-independent metagenomes were calculated after rarefying them (Fig. S1) to avoid bias due to different sequencing depths [63]. Observed and Shannon diversity were significantly higher in co-cultures than in culture-independent metagenomes, whereas inverse Simpson diversity showed no statistical difference between the two sample types (Wilcoxon rank sum test with *P*<0.001) (Fig. 1b). Nonetheless, taxonomic identity of the most abundant bacteria in both types of metagenomes was conserved. The co-culture and culture-independent metagenomes shared 1371 OTUs, which accounted for 94 % of the total composition of these metagenomes (Fig. 1c). The main difference between the two samples was the distribution of bacterial taxa. While *Nostocales* was the most abundant bacterial order in both types of metagenomes, the non-cyanobacterial orders were, on average, more abundant in the co-culture metagenomes (Fig. S2). These results indicate that the co-culture strategy previously used to investigate symbiotic and other tightly assembled communities [5,64] provides an adequate representation of the bacterial diversity present inside cycad coralloid roots.

**Fig. 2.**
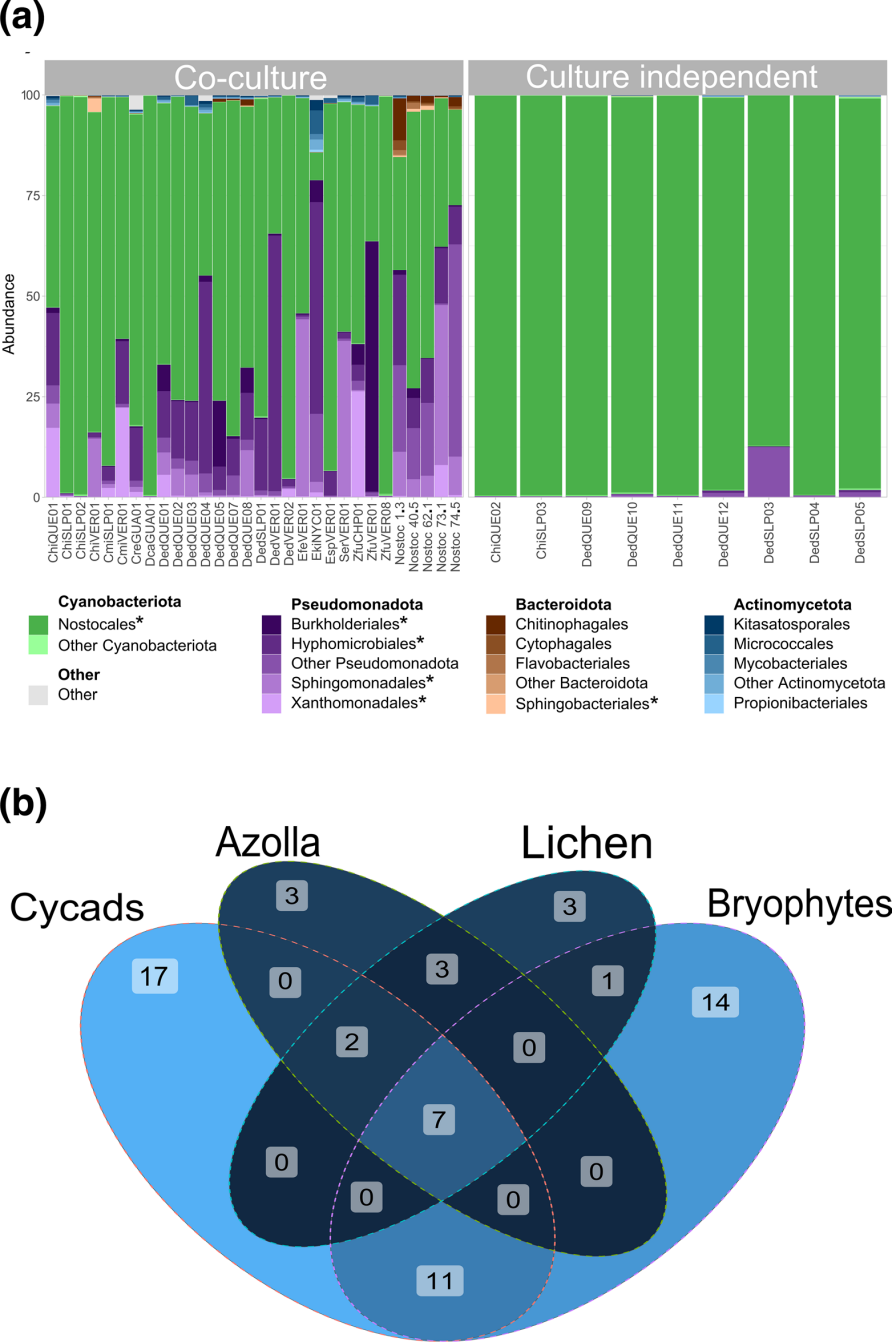
Taxonomic analysis of cycad coralloid root symbiotic communities and in other symbiotic systems. (**a**) Symbiotic communities from direct extractions were consistently dominated by *Nostocales* cyanobacteria, whereas co-cultures were more diverse. The *Pseudomonadota* fraction of these communities was particularly enriched. (**b**) Seven taxonomic orders [marked in (a) plus *Caulobacter*] from the core taxa (present in at least 80 % of all samples) obtained from all metagenomes were not only found in other coralloid root symbiotic communities, but also in other symbiotic systems. A full description of orders in the Venn diagram is given in Table S3.

Taxonomic assignment of reads in all metagenomes, irrespective of how they were generated, revealed a bacterial composition mostly composed of the phyla *Cyanobacteriota*, *Pseudomonadota*, *Actinomycetota* and *Bacteroidota*. Of these, *Cyanobacteriota* was dominant in 82 % of the samples, including all metagenomes obtained from direct extractions, while *Pseudomonadota* was the dominant taxon in the remaining 18 %, as well as the second most abundant in all cyanobacteria-dominated metagenomes. The cyanobacterial fraction consisted of *Nostocales*, while the remaining phyla consisting of *Pseudomonadota*, *Actinomycetota* and *Bacteroidota* was more diverse, both in composition at the order level and in abundance (Fig. 2a). Overall, a bacterial core composed of 29 bacterial orders was found to be present in at least 80 % of all samples (Table S1).

### Multiple and different symbiotic hosts share a common bacterial core

The identified bacterial orders herein have been previously found in symbiotic communities from cycads [5,8]. Furthermore, seven bacterial orders, namely *Nostocales, Hypomicrobiales, Caulobacterales, Sphingomonadales, Burkholderiales, Xanthomonadales* and *Sphingobacterales,* have been also reported in symbiotic communities from bryophytes [21], lichens [35,38] and various *Azolla* species [37] (Fig. 2b). Thus, our results show that a conserved bacterial core is shared between cycads and other symbiotic hosts.

### Symbiotic *Nostocales* have a polyphyletic origin

To better understand the cyanobacterial diversity revealed by metagenomics in the previous section, we aimed to reconstruct the phylogenomic relationships of *Nostocales* cycad cyanobionts. At least one cyanobacterial MAG from each metagenome was assembled using a custom pipeline for short- and long-read sequences. Amongst these, four metagenomes obtained from *Ceratozamia hildae* and *Dioon edule*, i.e. two co-cultures (ChiQUE01 and DedVER01) and two direct extractions (ChiSLP03 and DedQUE12), contained two distinct and thus sympatric cyanobacterial MAGs, which had previously been suggested based on 16S analysis [19] and confirmed herein. Due to these co-occurring organisms, the total number of recovered *Nostocales* MAGs was higher than the number of co-cultures (43 and 39, respectively; Table 2). We then assigned species identity to all MAGs using the genome taxonomy database toolkit (GTDB-Tk) [53] (Table S3), followed by reconstruction of a *Nostocales* phylo(meta)genomic tree using a set of 251 conserved cyanobacterial markers [57]. The resulting tree was rooted with *Chlorogloea* sp. CCALA 695, a *Chroococcidiopsidales* cyanobacterium located in the *Nostocales* sister clade [65].

**Table 2.** Symbiotic MAGs obtained

MAG name	Original metagenome	Genome size (Mb)	CompleteBUSCOs (%)	GC (%)	Accession orBioproject
*Nostoc* sp. ChiQUE01a MAG	ChiQUE01	8.3	99	41.2	JAVRBL000000000*
*Nostoc* sp. ChiQUE01b MAG	ChiQUE01	9.7	98.7	41.45	JAVRBM000000000*
*Nostoc* sp. ChiQUE02 MAG	ChiQUE02	8.7	99.5	41.91	JAVRBN000000000*
*Nostoc* sp. ChiSLP01 MAG	ChiSLP01	9.3	99.3	41.05	JAVRBO000000000*
*Nostoc* sp. ChiSLP02 MAG	ChiSLP02	8.6	99.5	40.88	JAVRBP000000000*
*Nostoc* sp. ChiSLP03a MAG	ChiSLP03	8.6	99.2	41.28	JAVRBQ000000000*
*Dendronalium* sp. ChiSLP03b MAG*	ChiSLP03	8.1	96.4	41.81	JAVRBR000000000*
*Nostoc* sp. ChiVER01 MAG	ChiVER01	8.3	99.6	41.1	JAVRBS000000000*
*Nostocsp*. CmiSLP01 MAG	CmiSLP01	9.4	99.4	40.99	JAVRBT000000000*
*Nostoc* sp. CmiVER01 MAG	CmiVER01	8.1	99.7	41.1	JAVRBU000000000*
*Nostoc* sp. CreGUA01 MAG	CreGUA01	7.5	97.2	41.15	JAVRBV000000000*
*Nostoc* sp. DcaGUA01 MAG	DcaGUA01	7.9	99.5	41.22	JAVRBW000000000*
*Nostoc* sp. DedQUE01 MAG	DedQUE01	7.8	98.9	41.24	JAVRBX000000000*
*Nostoc* sp. DedQUE02 MAG	DedQUE02	8.6	99.3	41.5	JAVRBY000000000*
*Nostoc* sp. DedQUE03 MAG	DedQUE03	8.3	97	41.39	JAVRBZ000000000*
*Nostocsp*. DedQUE04 MAG	DedQUE04	9.0	99.1	41.47	JAVRCA000000000*
*Nostoc* sp. DedQUE05 MAG	DedQUE05	8.3	99.3	41.45	JAVRCB000000000*
*Nostoc* sp. DedQUE07 MAG	DedQUE07	7.9	98.1	41.48	JAVRCC000000000*
*Nostoc* sp. DedQUE08 MAG	DedQUE08	8.8	99.2	41.44	JAVRCD000000000*
*Nostoc* sp. DedQUE09 MAG	DedQUE09	8.2	98.8	41.67	JAVRCE000000000*
*Aulosira* sp. DedQUE10 MAG*	DedQUE10	8.5	98.5	41.42	JAVRCF000000000*
*Nostoc* sp. DedQUE11 MAG	DedQUE11	7.9	99.4	41.24	JAVRCG000000000*
*Nostoc* sp. DedQUE12a MAG*	DedQUE12	8.1	95.4	41.03	JAVRCH000000000*
*Nostoc* sp. DedQUE12b MAG*	DedQUE12	7.6	96.6	41.53	JAVRCI000000000*
*Nostoc* sp. DedSLP01 MAG	DedSLP01	8.9	99.5	41.03	JAVRCJ000000000*
*Nostoc* sp. DedSLP03 MAG	DedSLP03	9.1	99.4	41.32	JAVRCK000000000*
*Nostoc* sp. DedSLP04 MAG	DedSLP04	8.2	99.2	41.45	JAVRCL000000000*
*Nostoc* sp. DedSLP05 MAG	DedSLP05	8.8	99.5	40.97	JAVRCM000000000*
*Aulosira* sp. DedVER01a MAG	DedVER01	8.2	99.4	41.32	JAVRCN000000000*
*Nostoc* sp. DedVER01b MAG	DedVER01	7.8	99.4	41.12	JAVRCO000000000*
*Nostoc* sp. DedVER02 MAG	DedVER02	7.7	99.4	41.12	JAVRCP000000000*
*Nostoc* sp. EfeVER01 MAG	EfeVER01	7.3	98.7	41.61	JAVRCQ000000000*
*Nostoc* sp. EkiNYC01 MAG	EkiNYC01	9.1	99.3	41.61	JAVRCR000000000*
*Nostoc* sp. EspVER01 MAG	EspVER01	7.0	99.1	41.59	JAVRCS000000000*
*Nostoc* sp. SerVER01 MAG	SerVER01	8.1	99.4	41.27	JAVRCT000000000*
*Aulosira* sp. ZfuCHP01 MAG	ZfuCHP01	8.3	99.5	41.32	JAVRCU000000000*
*Aulosira* sp. ZfuVER01 MAG	ZfuVER01	8.3	99.5	41.32	JAVRCV000000000*
*Nostoc* sp. ZfuVER08 MAG	ZfuVER08	8.2	99.2	40.68	JAVRCW000000000*
*Nostoc* sp. 1.3 MAG	Nostoc 1.3	8.3	90.8	41.51	1 325 816†
*Nostoc* sp. 40.5 MAG	Nostoc 40.5	8.3	99.6	41.56	1 325 815†
*Nostoc* sp. 62.1 MAG	Nostoc 62.1	8.4	99	41.53	1 325 813†
*Nostoc* sp. 73.1 MAG	Nostoc 73.1	8.5	99.2	41.5	1 325 811†
*Nostoc* sp. 74.5 MAG	Nostoc 74.5	8.7	99.3	41.29	1 325 814†

a *NCBI gGenbBank accession number.

b †JGI Seq Project ID.

**Fig. 3.**
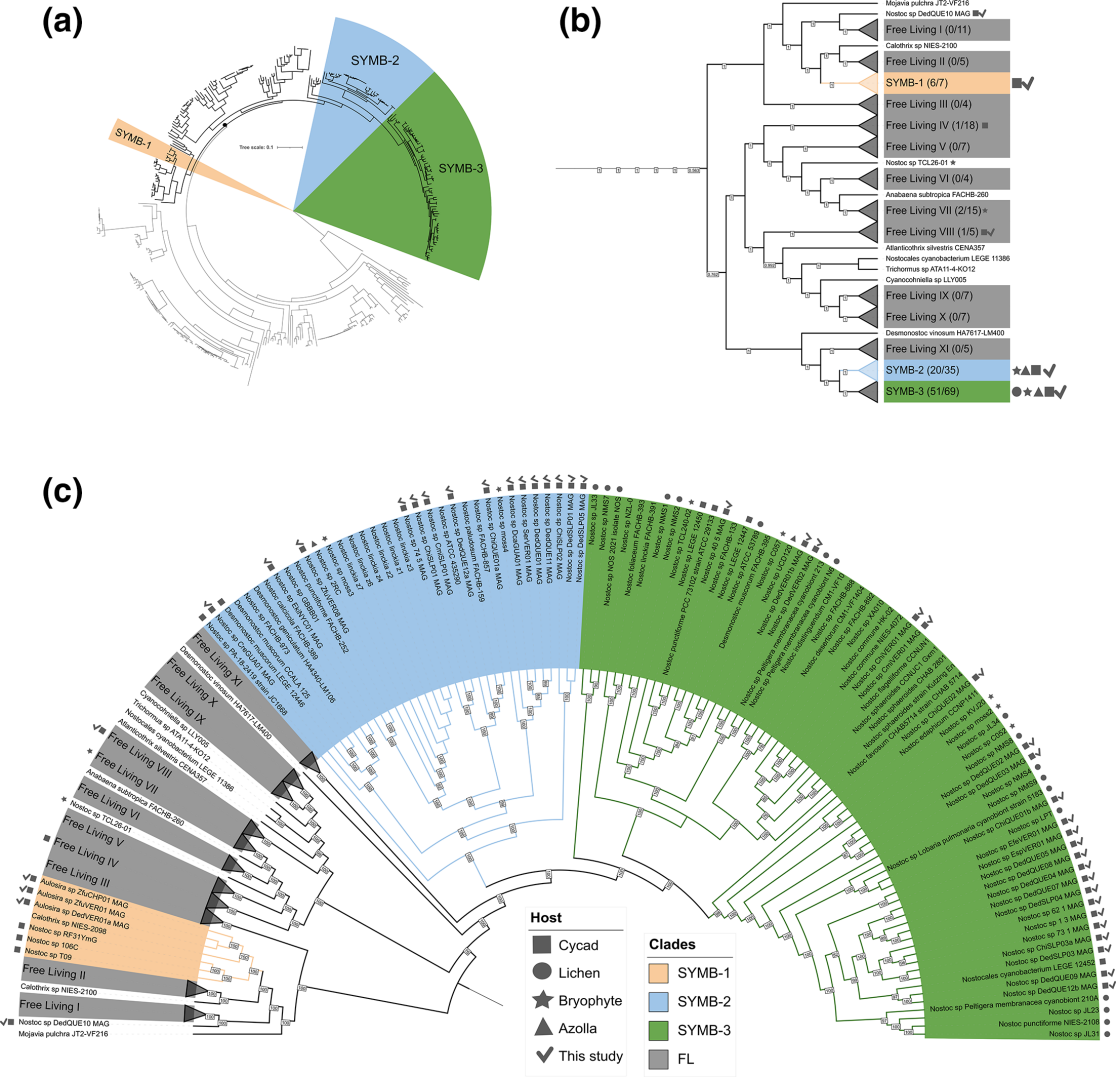
Phylogenomic reconstruction of *Nostocales* cyanobionts. (**a**) Phylogenomic tree of 372 *Nostocales* genomes made with a set of 251 single-copy cyanobacterial genes and rooted with *Chlorogloea* sp*.* CCALA 695. The monophyletic origin of most cyanobionts is marked with a dot. (**b**) Cladistic distribution supported by ANI similarity of the 209 genomes in the super-clade. The number of genomes per clade (in parentheses) and host (forms) is provided. The symbiotic clades SYMB-1, 2 and 3 are marked in yellow, blue and green, respectively. Free living clades are numbered consecutively from top to bottom (FL-I to FL-XI) and coloured in grey. (**c**) Zoom in of the super-clade, with the symbiotic clades. Only bootstrap values >75 are shown. See main text for a detailed description.

Inspection of our *Nostocales* phylogeny revealed that all facultative symbiotic cyanobionts from this study, and from the database, have a monophyletic origin (Fig. 3a). A super-clade containing interspersed cyanobionts was then subdivided into sub-clades, as defined by an analysis using ANI (Fig. S3). In total, 14 clades, three of which were found to contain 77 of the 83 species annotated as symbiotic (92 %), including 40 of the 43 MAGs obtained in this study (95 %), were identified. These clades were named SYMB-1, 2 and 3, while all remaining clades containing mostly (reported) free-living species, were named FL I–XI (Fig. 3b, c). The remaining 5 % of species with a symbiotic origin contained in clades FL I–XI include two species obtained here after direct extraction, i.e. *Aulosira* sp. DedQUE10 MAG from *Dioon edule*, an outgroup of FL-I, and *Dendronalium* sp. ChiSLP03 MAG from *Ceratozomia hildae,* located inside FL-VIII. Further members include *Nostoc cycadae* WK-1, obtained from *Cycas revoluta* in Japan [66] and located inside FL-IV; *Nostoc* sp. TLC26-01 isolated from the bryophyte *Leiosporoceros dussii* in Panama [67], and found as an outgroup of clade FL-VI; and *Nostoc* sp. moss5 and moss6 isolated from mosses in Norway [25], and located inside FL-VII.

The SYMB-1 clade consists of *Aulosira* sp. DedVER01a MAG, from *Dioon edule; Aulosira* sp. ZfuVER01 MAG and ZfuCHP01 MAG, from *Zamia furfuracea* (different locations); *Nostoc* sp. T09, from a Mexican *Dioon caputoi; Nostoc* 106C and RF31YmG from Mexican *Dioon merolae,* previously reported by us [5]; and one species annotated as free-living, *Calothrix* sp. NIES-2098 [68], but for which available metadata are scarce. This is the least populated symbiotic clade, and although we and others have noted its existence before [5,8], the newly generated data of this study consolidate it as a cycad-specific symbiotic lineage that evolved independently of the better known *Nostoc* lineages of SYMB-2 and SYMB-3. Since no Australian cyanobionts were found in this clade, this was the only SYMB clade that is exclusively made up of American cyanobionts.

Clade SYMB-2 contained an even distribution of cyanobionts and free-living species with 18 out of 30 cyanobacteria isolated from symbiotic hosts (Fig. 3b). Symbiotic species mostly from cycads were found herein, with the exception of *Nostoc* sp. 2RC, which was isolated from *Azolla pinnata* from the USA [27], and *Nostoc* sp. moss 3 and 4, which were isolated from *Pleurozium schreberi* mosses from a Swedish boreal forest [25]. Previously reported cycad cyanobionts include *Nostoc* sp. PA-18-2419 strain JC1668 from *Zamia pseudoparasitica* WP in Panama [8], and *Desmonostoc muscorum* LEGE 12446, isolated from a *Cycas revoluta* in a BG in Portugal [69]. The remaining 15 strains were obtained from cycads (this study), four of which were from BG, *Nostoc* sp. EkiNYC01 MAG, isolated from *Encephalartos kisambo* in the New York Botanical Garden; *Nostoc* sp. SerVER01 MAG, from *Stangeria eriopus* in the Francisco Javier Clavijero BG, Veracruz, Mexico; and two strains from a PC, *Nostoc* sp. CreGUA01 MAG and *Nostoc* sp. DcaGUA01 MAG, isolated from *Cycas revoluta* and *Dioon caputoi*, respectively. The remaining 11 genomes were obtained from WP from Mexico and Australia and included three species obtained from culture-independent metagenomes: *Nostoc* sp. DedQUE12a, *Nostoc* sp. DedQUE11 and *Nostoc* sp. DedSLP05. Taken together, the composition of this clade confirms it as a cycad symbiotic lineage, but also with signs of symbiotic plasticity due to the grouping of FL organisms and those from other hosts.

The SYMB-3 clade contained most of the symbiotic strains, with 70 % of the genomes reported as isolated from diverse hosts, including cycads, lichens, bryophytes and *Azolla*. *Nostoc* sp. UCD 120, from *Azolla caroliniana*, was the only representative from this host [27]. From bryophytes, the representatives were *Nostoc* sp. TCL240-02 from *Leiosporoceros dussii* [67], *Nostoc* sp. moss 2 from *Pleurozium schreberi*, and *Nostoc* sp. KVJ20 [25] and *Nostoc* sp. C052 [67], both from *Blasia pusilla*. From cycads, 27 genomes were included in this clade, of which 23 are from this study. These included five species isolated from direct culture-independent extractions, namely *Nostoc* sp. ChiSLP03a MAG, *Nostoc* sp. DedQUE09 MAG, *Nostoc* sp. DedQUE12b MAG, *Nostoc* sp. DedSLP03 MAG and *Nostoc* sp. DedSLP04 MAG. The cycad symbionts from the literature that fell in this clade were *Nostoc* sp. LEGE 12450, *Nostoc* sp. LEGE 12447, *Nostocales cyanobacterium* LEGE 12452 (LEGE collection, Portugal) and the well-characterized *Nostoc punctiforme* PCC 73102 [70]. This last species was isolated from an Australian *Macrozamia* cycad and exhibits symbiotic plasticity, capable of forming symbiotic relationships with other hosts. Additionally, four of the five Australian MAGs were found in this clade, namely *Nostoc* sp. 40.5 MAG (*Macrozamia communis*), 62.1 (*Macrozamia mountperriensis*), 1.3 (*Bowenia serrulata*) and 73.1 (*Macrozamia serpentina*). Interestingly, all 18 lichen symbionts reported in the literature were in this clade.

### The symbiotic *Nostocales* have large and open pangenomes

**Fig. 4.**
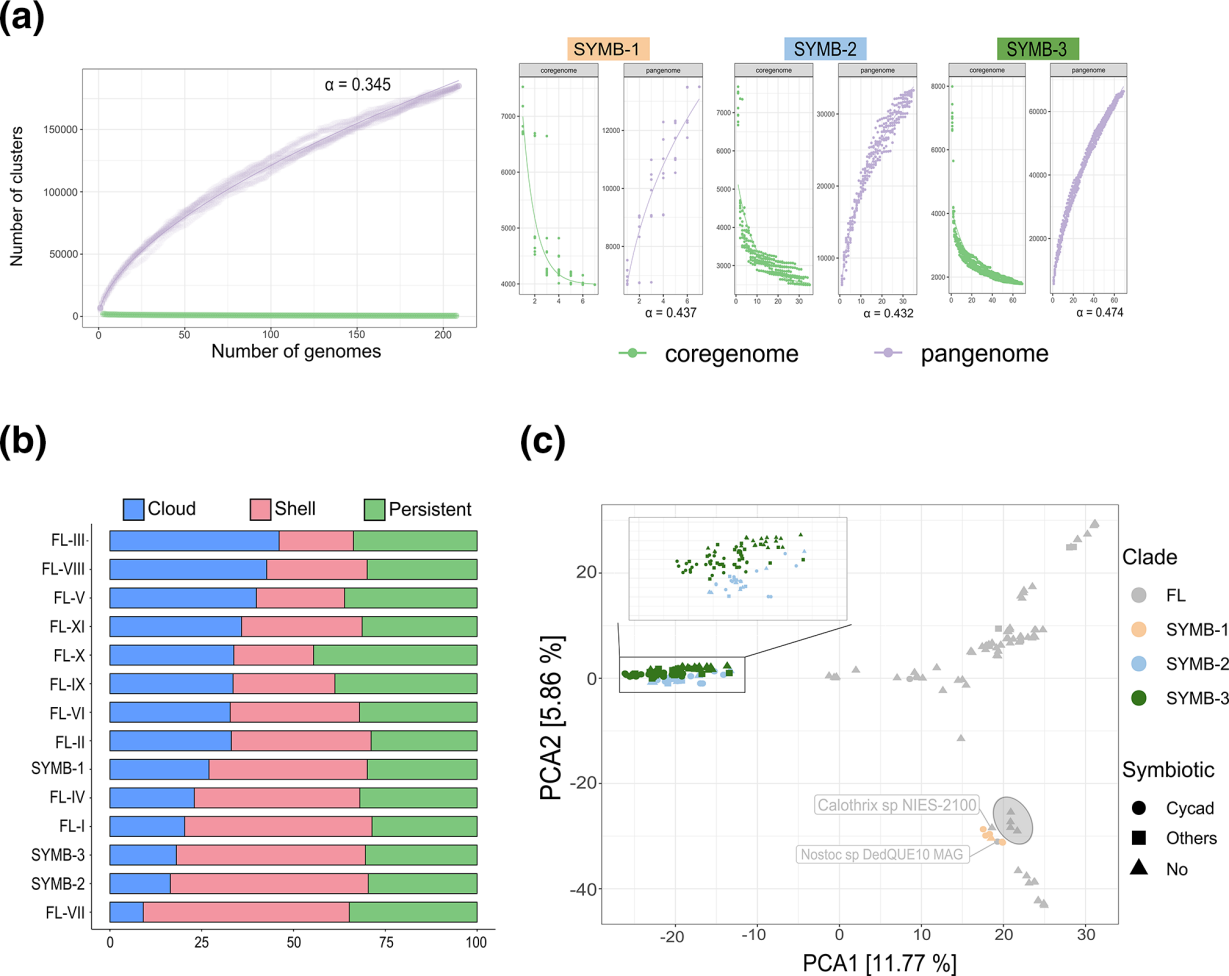
Genomic analysis of *Nostocales* symbiotic clades. (**a**) Pangenome and core genome curves of the super-clade and the three symbiotic clades. The shape and Heap alpha value for the three symbiotic clades indicate that they have an open pangenome. (**b**) Proportion of genes in each clade found in the persistent, shell and cloud partitions. Symbiotic clades SYMB-2 and SYMB-3 have a higher proportion of their genomes in the shell partition, whereas SYMB-1 is evenly split between the three categories. (**c**) Principal components analysis of the pangenomes showing that SYMB-2 and SYMB-3 are similar, whereas SYMB-1 is more closely related to its sister FL-II clade (grey circle).

Using the phylogenomic reconstructions as a guide, we then asked whether SYMB clades may have functional phylogenetic signals underlying their symbiotic lifestyles. Genome sizes throughout the FL clades were found to range from 3.9 to over 10 Mb, with an average of 7.3 Mb. Except for *Nostoc sphaeroides* strain Kutzing En, all genomes in the SYMB clades had a genome size >7 Mb, with an average of 8.5 Mb (Fig. S4). The relationship between genome size and gene content within the SYMB clades was the same as in the FL clades (Fig. S5), suggesting that no genome streamlining occurs in facultative cyanobionts, in contrast to what has been observed in obligate cyanobionts (Flores et al., 2022), and, if any, a functional symbiotic signal would not be obvious. To further investigate this, we performed a comprehensive pangenomic analysis. First, we assessed whether the *Nostocales* in the super-clade, and in the three symbiotic clades, had an open or closed pangenome. Open pangenomes are typical of species that can colonize different niches, whereas closed pangenomes are indicative of species restricted to one environment [71]. An alpha value of <1 from the power law was obtained for the entire *Nostocales* super-clade, indicating an open pangenome [72] consistent with the multiple lifestyles characteristic of these species [73]. Similarly, the symbiotic clades exhibited an open pangenome, which is consistent with a facultative symbiotic lifestyle [28,74] (Fig. 4a).

### Pangenomic analysis revealed that each symbiotic clade has distinct genomic features

We measured the average percentage of gene families from the genetic pool of each clade that belongs to the persistent (conserved in most genomes), shell (conserved in some genomes) and cloud (present only in one of few genomes) partitions [75]. In the symbiotic clades SYMB-2 and SYMB-3, most gene families were classified as part of the shell partition of the pangenome, whereas SYMB-1 was more evenly distributed between the cloud, shell and persistent partitions (Fig. 4b). The small number of gene families from the cloud partition in the symbiotic clades SYMB-2 and 3 indicates that many of the genomes in these clades share gene families. A principal components analysis (PCA) plot of the pangenome revealed that, indeed, these two clades have a high degree of similarity between themselves but are different from the rest of the species in the super-clade, including those from SYMB-1 (Fig. 4c). Zooming in on the SYMB-2 and 3 clades revealed that there was no grouping based on lifestyle, symbiotic vs non-symbiotic, or host, cycad vs other hosts (Fig. 4c). The PCA also revealed that genomes in SYMB-1 are in a different quadrant than those from SYMB-2 and 3, which indicated that they differ in genome content. SYMB-1 genomes are closer to those in its sister clade FL-II (see Fig. 3c) and had two extra genomes intermixed in their midst: *Calothrix* sp. NIES-2100 and *Aulosira* sp. DedQUE10 MAG, the latter being a cyanobiont from cycads (Fig. 4c). However, an analysis of all cycad genomes revealed that *Aulosira* sp. DedQUE10 MAG, along with other cyanobionts isolated from cycads, share just a few shell and cloud gene families that are conserved in all the cyanobiont genomes from the SYMB clades, which supports the proposed phylogenetic grouping (Fig. S6). This suggests that the cyanobionts outside of the SYMB clades are outliers. Furthermore, this analysis also indicates that, in contrast to what was observed in the taxonomic analysis, no differences were observed between Australian and American cyanobionts, based on geography.

### Genomes from the symbiotic clades have a conserved functional profile without cyanobiont-specific genes

**Fig. 5.**
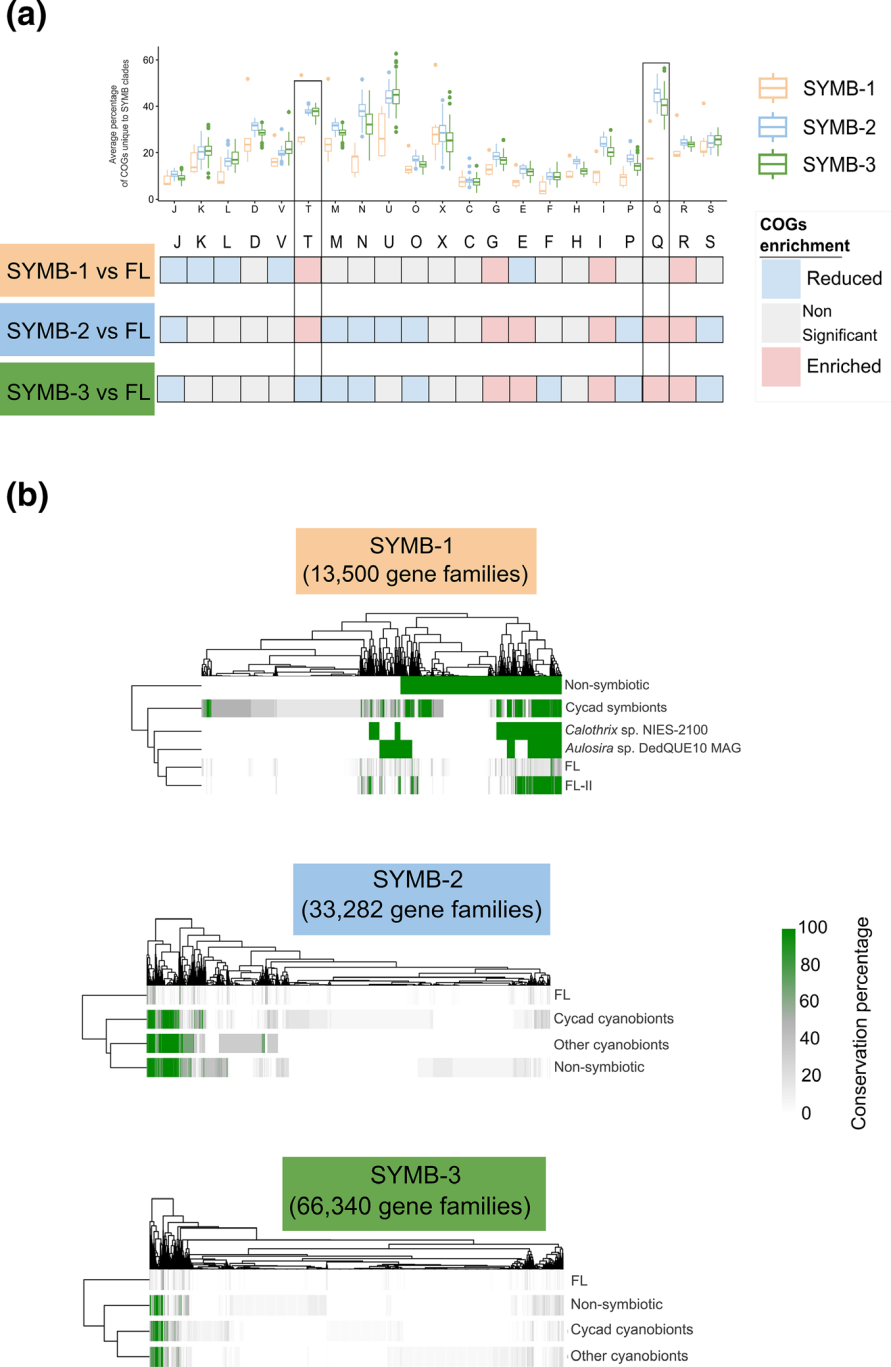
Whole-clade analysis of potential functional symbiotic genomic signals. (**a**) Functional COG categories enrichment analysis of genes from SYMB clades compared to all FL clades. Average percentage of genes in each COG group per clade that are unique to the SYMB clades is shown at the top. Statistically significant reductions or enrichments are shown at the bottom. These were obtained by taking all genomes from each SYMB clade, calculating the average proportion of the coding genome dedicated to each COG category and comparing it to the average obtained from all FL clades (*P*<0.001; Kruskal–Wallis rank sum test). COG groups T and Q are the only enriched categories with more than <30 % unique genes (box). (**b**) Comparative analysis of all gene families from the shell and cloud partitions in each SYMB clade against the FL clades. Genomes from the SYMB clades were divided into three categories, Cycad cyanobionts, Other cyanobionts and non-symbiotic. All genomes not in an SYMB clade were allocated to the FL group. For SYMB-1, FL genomes were further sub-divided to account for closely related genomes to SYMB-1. Gene families were coloured by prevalence, with green tones representing conservation in more than 60 % of the genomes in each group. This revealed that conserved gene families are shared by all genomes from each SYMB clade, including non-symbiotic genomes. COG functions are as follows: [J] Translation, ribosomal structure and biogenesis; [K] Transcription; [L] Replication, recombination and repair; [D] Cell cycle control, cell division, chromosome partitioning; [V] Defence mechanisms; [T] Signal transduction mechanisms; [M] Cell wall/membrane/envelope biogenesis; [N] Cell motility; [U] Intracellular trafficking, secretion and vesicular transport; [O] Posttranslational modification, protein turnover, chaperones; [X] Mobilome: prophages, transposons; [C] Energy production and conversion; [G] Carbohydrate transport and metabolism; [E] Amino acid transport and metabolism; [F] Nucleotide transport and metabolism; [H] Coenzyme transport and metabolism; [I] Lipid transport and metabolism; [P] Inorganic ion transport and metabolism; [Q] Secondary metabolite biosynthesis, transport and catabolism; [R] General function prediction only; [S] Function unknown.

Based on the realization that the pangenome of cyanobionts seems to be different from that of FL cyanobacteria, a general functional classification of Clusters of Orthologous Genes, or COGs [76], was performed. With this, we were hoping to identify changes related to functional traits potentially underlying the symbiotic lifestyle of the organisms in the SYMB clades. COG functions were obtained for all genomes in the super-clade, and the average proportion of the genome dedicated to each function was calculated (top, Fig. 5a). This allowed us to compare symbiotic clades with FL clades in a normalized fashion (bottom, Fig. 5a). Overall, these combined analyses revealed a differential pattern of statistically significant functional enrichments and reductions specific to the SYMB clades (Wilcoxon rank sum test with *P*<0.001).

In all three SYMB clades, COG groups G (Carbohydrate transport and metabolism), I (Lipid transport and metabolism) and R (General function) were enriched, and group J (Translation, ribosomal structure and biogenesis) was reduced. In SYMB-1, groups K (Transcription), L (Replication, recombination and repair) V (Defence mechanisms) and E (Amino acid transport and metabolism) were reduced. The only enrichment was observed in group T (Signal transduction mechanisms), which was also observed in clade SYMB-2. Clades SYMB-2 and SYMB-3, again, were similar, with both showing enrichments in COG groups Q (Secondary metabolite biosynthesis, transport and catabolism) and E (Amino acid transport and metabolism). Species in these clades also had similar gene reductions, having on average a lesser proportion of their genomes dedicated to COG functional groups M (Cell wall/membrane/envelope biogenesis), N (Cell motility), O (Posttranslational modifications, protein turnover and chaperons), P (Inorganic ion transport and metabolism) and S (Unknown function). These results indicate that while general functional traits were found for all three SYMB clades, SYMB-1 has a different profile than SYMB-2 and 3. This is consistent with what was observed in the PCA in Fig. 3c), in which we observed that SYMB-2 and SYMB-3 have shared gene families that differentiate them from the rest of the *Nostocales* in the super-clade, including SYMB-1. This supports the idea that the species in SYMB-1 employ different symbiotic mechanisms.

We checked how many of these functionally categorized genes were unique to the symbiotic clades. Interestingly, genomes from SYMB-1 had a lower percentage of unique genes than SYMB-2 and SYMB-3. This could be explained by the relatively sparse number of genomes available for SYMB-1 in comparison to SYMB-2 and 3. The grouping observed in the PCA (Fig. 3c) suggested that SYMB-1 genomes share many gene families with their sister FL-II clade. This suggests that differentiation between both clades has been recent and/or there are other factors at play, such as horizontal gene transfer (HGT). The SYMB-1 trend of having a lesser percentage of unique genes was more pronounced for the COG groups with the highest average percentage of unique genes in SYMB-2 and 3, namely COGs M, N, T and Q (top, Fig. 5a). Two of these COG groups, T in SYMB-1 and SYMB-2 and Q in SYMB-1 and SYMB-2, stand out, as they were the only two COGs with both enriched and unique genes. COG group T corresponds to signal transduction mechanisms that may contribute to the cyanobiont’s ability to recognize the host chemical signals, which is a necessary step in the establishment of symbiosis [25,77, 78]. The high number of genes found in COG group Q, which corresponded to secondary metabolism, indicated that natural products might play a more significant role in the symbiosis than previously thought, consistent with observations made on the uniqueness of metabolites in cyanobionts [5,14].

We then proceeded to undertake a targeted analysis of the gene families from the shell and cloud partitions per SYMB clade (13 500 for SYMB-1, 33 282 for SYMB-2 and 66 340 for SYMB-3). By doing so, we intended to identify a set of conserved genes families exclusive to, and prevalent in, symbiotic strains, whether from cycads or other symbiotic systems. However, no such group of gene families was found (Fig. 5b). While genomic composition of genomes from the SYMB-2 and 3 clades was clearly different from FL genomes, no clear differences were observed that differentiated symbiotic species from non-symbiotic species. This is consistent with what was found in the PCA (Fig. 3c), which prompted us to separate the genomes from FL and SYMB-1 into subgroups for further analysis. Specifically, the separation was done to differentiate FL-II, the SYMB-1 sister clade, and the two outliers, *Calothrix* sp. NIES-2100 and *Aulosira* sp. DedQUE10 MAG, from the rest of the genomes in FL clades. Furthermore, it should be noted that clade SYMB-1 only contains one free-living genome and therefore no generalization can be made about the results obtained from comparing it to the symbiotic genomes.

After detailed analysis we found that none of the sub-grouped genomes had a similar pattern to the one observed in SYMB-1 genomes, as was observed in cycad symbionts (Fig. S6), and thus supporting the idea that SYMB-1 genomes have a unique genomic composition. Yet, it is apparent that all genomes in the SYMB clades have a distinct pattern of functional enrichments/decrements (COG categories) that is related to the symbiotic lifestyle, most notably those related to production of natural products (COG group Q) and signal transduction (COG group T). None of these functions, however, were encoded in a set of ‘symbiosis genes’.

Taken together, the combined results from this and the previous subsection strongly suggest that at least two phylogenetically and functionally different lineages of facultative *Nostocales* cyanobionts exist. One of these lineages, containing all genomes in SYMB-2 and 3, is enriched in unique genes with functional annotations that are congruent with functions that have been found to be necessary for symbiosis, such as motility, signal transduction mechanisms and carbohydrate metabolism [25,28, 77, 78], as well as those necessary for biosynthesis of unique natural products, which are characteristic of cyanobionts [5,14]; and could play an active role in the symbiosis [79,80]. The remaining lineage, which includes all genomes from SYMB-1, has a unique enrichment/decrement pattern that does not seem to be driven by differential genes. Overall, we found that the ability of *Nostocales* to form facultative symbiosis is a complex trait that does not depend upon specific genes and could even be thought of as a gradient instead of a binary event, supporting the concept of symbiotic plasticity.

## Discussion

Despite the steady increase in the number of cyanobiont genomes in public databases, whether from cycads or other hosts, their phylogenomic analysis has been limited to the analysis of a few selective genomes in each study, resulting in inconsistencies regarding the number of symbiotic clades and the species therein [5,8, 24, 25, 26]. This has hampered the elucidation of both general, and cycad-specific, cyanobacterial symbiotic signals, especially in elucidating cyanobacterial symbiotic adaptations in both aquatic and terrestrial environments [81]. Furthermore, it is also unknown how big of a role the associated bacterial community may play in the symbiosis process, and whether their composition is dictated by geography [8,21], cyanobiont selection [5,82], host selection [37] or a combination of all these factors. Here, we present a comprehensive phylogenomic analysis of facultative cyanobionts, coupled with the taxonomic meta-analysis of their associated bacterial community, directly from cycad coralloid roots and in culture. We thereby provide a general overview of the diversity found in a variety of environments, clarifying the apparent cladistic distribution of symbiotic *Nostocales* species.

Taxonomic analysis of symbiotic communities in coralloid roots from American [5,6, 8] and Asian [7,83] cycads has revealed a diverse bacterial community mostly dominated by *Cyanobacteria* and *Pseudomonadota*, which have been reported in other symbiotic systems [28]. Our metagenomes were also dominated by *Cyanobacteria* and *Pseudomonadota,* with *Bacteroidota* or *Actinomycetota* being the third mot abundant phyla in most of the samples, the former being consistently found in Australian cycads and more sporadically in American cycads (Fig. 2a). The ratio between these two phyla, however, was dependent on whether the communities were sequenced directly after biomass extraction from coralloid roots or after co-culturing (Fig. S2). While the relative abundance of *Nostocales* in culture-independent metagenomes was always >80 %, their abundance in co-culture varied widely (Fig. 2a). Given that co-culture conditions are not reflective of the coralloid root inner environment, these changes are in line with previous studies that reported a similar shift in cyanobacterial dominance between the coralloid roots’ endosphere and their surrounding soil [6]. Interestingly, the taxonomic identity of dominant taxa was also consistent between samples from both extraction methodologies, despite the different nutritional and environmental conditions that communities in co-culture are exposed to compared to the coralloid root, indicating that once formed, symbiotic communities remain stable (Fig. 1c). Composition was also similar regardless of sampling location, with Australian cycads having a taxonomic composition comparable to their American counterparts. Even more so, this study confirms that seven bacterial orders, i.e. *Nostocales, Hypomicrobiales, Caulobacterales, Sphingomonadales, Burkholderiales, Xanthomonadales* and *Sphingobacterales*, can be found in cycads and other symbiotic systems [21,35,38] (Fig. 2b). This degree of universal conservation implies selection [84] that might be based in supporting functional roles [37,82].

Cyanobiont-specific analysis revealed three paraphyletic clades that contained 95 % of all symbiotic species (Fig. 3). All clades included free-living species as well, but, to our surprise, their genomic composition, based on ANI similarity (Fig. S3) and pangenomic analysis (Figs3c  5a), was indistinguishable from their symbiotic counterparts, although it should be noted that some of the so-called free living species might also be capable of forming a symbiotic relationship and they just happened to be isolated from a non-symbiotic niche. At first glance, this observation may be in line with the idea that cyanobiont genomes lose symbiosis genes after they are removed from their symbiotic systems and maintained in laboratory conditions [27], similar to what has been observed in *Rhizobium* [85]. However, the MAGs obtained from direct metagenomes were not significantly different from those obtained from co-cultures from the same clade (Fig. S3). Moreover, pangenomic analysis revealed that SYMB-2 and SYMB-3 genomes were different to all other genomes, including those from SYMB-1 (Fig. 4c). This indicates that at least two divergent lineages of symbiotic *Nostocales* have evolved. Even though genomes from SYMB-2 and SYMB-3 are phylogenomically related, the fact that only the latter contains lichen cyanobionts (with the characteristic features of this tripartite association) (Fig. 3b) suggests that they might have unique symbiosis mechanisms. Previous efforts aimed at finding shared genomic features in cyanobionts have used genomes that, considering the phylogeny presented here, are not phylogenetically related [5,25], which may explain why previously reported symbiosis genes have been found outside cyanobionts [27].

Even though cyanobacteria may use different mechanisms to adopt a symbiotic lifestyle, they still share certain general functional features. These include an increment of the coding genome fraction dedicated to COG functions related to lipid and carbohydrate metabolism (Fig. 5b), which suggests that these are necessary for the symbiotic lifestyle, as functional enrichment has been linked to adaptation [81,86]. This is consistent with previous findings [25,78] that have found that these functions are necessary for symbiosis. Genomes from SYMB-2 and SYMB-3 were also found to be enriched in genes related to amino acids and secondary metabolism (Fig. 5b) which have also been reported to be characteristic of cyanobionts [5,25]. More than 40 % of the genes assigned to the latter function, namely secondary metabolism, were only found in symbiotic clades, which further emphasizes the uniqueness of natural products produced by symbiotic cyanobacteria, something that has been recently highlighted (Fig. 5a) [14]. Other COG categories with a high proportion of unique genes were those dedicated to intracellular trafficking, motility and signal transduction mechanisms (Fig. 5a). The importance of these mechanisms in cyanobiont symbiosis has been previously reported [25,27, 28]. Interestingly, genomes from clade SYMB-1 contained a lower fraction of unique genes from these categories than those from SYMB-2 and 3 (Fig. 5a), in line with what was observed in the pangenomic analysis, namely that based on composition, genomes from SYMB-1 are closer to FL-II than SYMB-2 and 3 are to their sister clade FL-XI (Fig. 3c).

The present study, to the best of our knowledge at the time of writing, has quadrupled the number of available cycad cyanobiont’ genomes, and nearly doubled the overall number of cyanobiont genomes available. Despite this, the cycad-specific clade SYMB-1 is underrepresented (Fig. 3c). This study suggests that cyanobionts are not dependent on a specific set of conserved symbiosis genes. Nonetheless, general functional traits are shared by all SYMB genomes. Furthermore, they also share a similar bacterial community. While cyanobionts were clustered by clade rather than geography, taxonomic identity of dominant associated bacteria remained constant. The function of these communities, and the specific mechanisms that different cyanobiont and their hosts use to establish the transition to a symbiotic state, are yet to be elucidated. This study provides researchers with a solid footing to further investigate the establishment of symbiosis, both in the association of cyanobionts with the critically endangered cycads, and other eukaryotic hosts.

## supplementary material

10.1099/mgen.0.001207 Uncited Table S1.

10.1099/mgen.0.001207 Uncited Fig. S2.

## Abbreviations

ANI: average nucleotide identity
BNF: biological nitrogen fixation
COG: Cluster of Orthologous Genes
MAG: metagenome assembled genome
OTU: operational taxonomic unit
PCA: principal components analysis
