# Supplementary material for: Phylometagenomics of cycad coralloid roots reveals shared symbiotic signals
Source: Microb Genom. 2024 Mar 7;10(3):001207. doi: 10.1099/mgen.0.001207 (PMC10999742; doi:10.1099/mgen.0.001207)
Supplement: Uncited Fig. S2. [file mgen-10-01207-s002.pdf]

Supplementary information

Phylometagenomics of cycad coralloid roots reveals shared symbiotic signals

Edder D. Bustos-Diaz[1,6], Arely Cruz-Perez[2], Diego Garfias-Gallegos[2], Paul M. D'Agostino [3], Michelle M. Gehringer[4], Angelica Cibrian -Jaramillo[2,5], and Francisco Barona-Gomez [1,6*]

Supplementary Figure 1. Rarefaction curves of metagenomes. Samples from Queretaro and San Luis Potosi obtained from both extraction methodologies before (A) and after (B) rarefying them.

Supplementary Figure 2. Culture independent metagenomes are dominated by Nostocales. Average relative abundance of the 15 more abundant bacterial orders in metagenomes from Queretaro and San Luis Potosi grouped by extraction methodology. Culture independent metagenomes are dominated by Nostocales while bacterial diversity in co-culture metagenomes is more evenly distributed.

Supplementary Figure 3. ANI similarity supports cladistic grouping. ANI similarity clustermap for all 209 Nostocales genomes used for pangenomic analysis. All genomes are grouped by clade and those annotated as symbiotic are marked with the same nomenclature used in figure 3B and 3C.

Supplementary Figure 4. All genomes in the symbiotic clades have similar sizes. The genome size of all 209 genomes used for pangenomic analysis revealed that while genome size in FL clades vary widely, it is mostly conserved between SYMB clades.

Supplementary Figure 5. All genomes have a linear relationship between size and gene content. All 209 Nostocales genomes used for pangenomic analysis have a linear correlation between size and gene content, expresses as coding sequences (CDS).

Supplementary Figure 6. Cycad cyanobiont genomes are grouped by clade. The pangenome of all cycad's cyanobiont revealed that these are grouped by clade rather than geography. Cyanobionts outside of the SYMB clades do not share homologous genes from the shell and cloud partitions (black lines) conserved in all the genomes from the SYMB clades.

Supplementary table 1. Bacterial core at Order level [provided in Excel spreadsheet]

Supplementary table 2. Bacterial orders from the bacterial core observed in this study (observed in at least 80 % of samples) and other facultative systems. The seven taxa found in all studies are remarked [provided in Excel spreadsheet]

Supplementary table 3. Extended MAG statistics [provided in Excel spreadsheet]

Supplementary table 4. *Nostocales* genomes used for phylogenomic analysis [provided in Excel spreadsheet]

Supplementary table 5. All genes used to generate the pangenome [provided in Excel spreadsheet]

Supplementary table 6. Genes from the pangenome with COG annotations [provided in Excel spreadsheet]

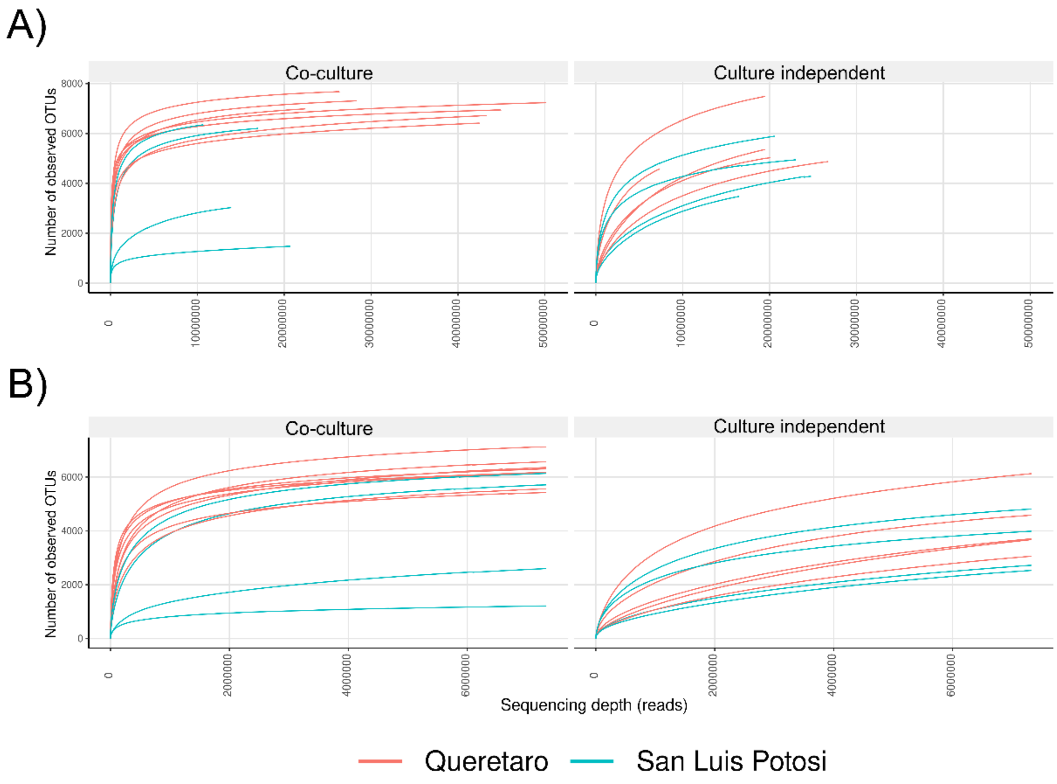

Supplementary Figure 1. Rarefaction curves of metagenomes. Samples from Queretaro and San Luis Potosi obtained from both extraction methodologies before (A) and after (B) rarefying them.

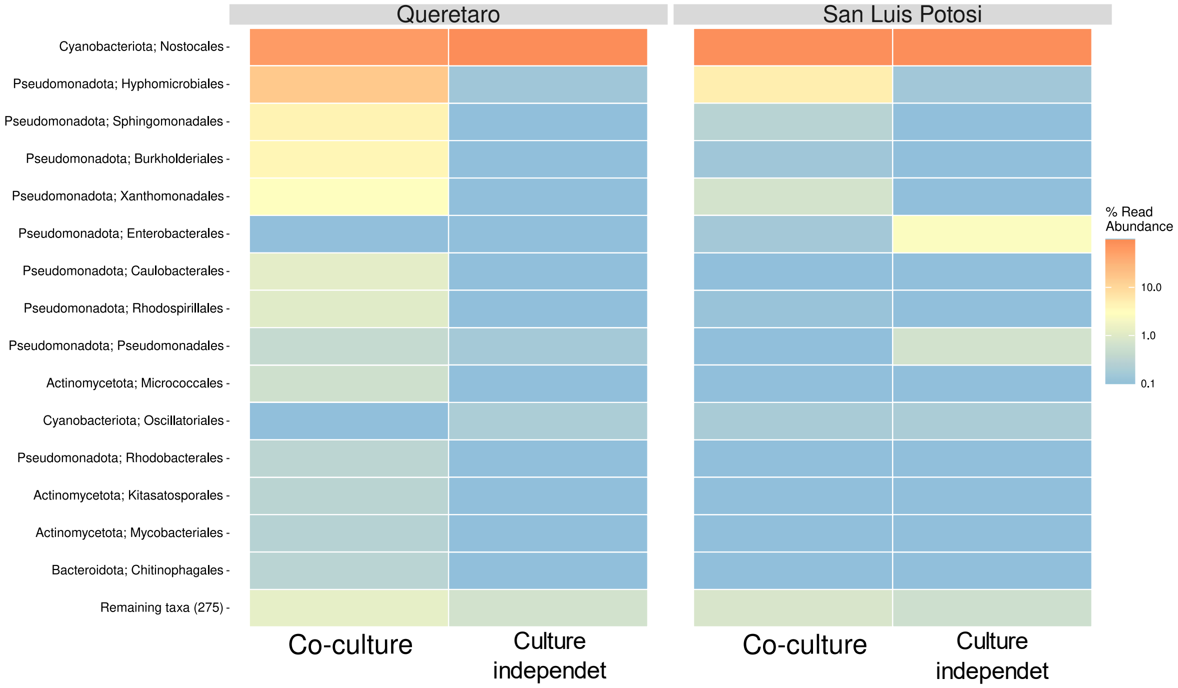

Supplementary Figure 2. Culture independent metagenomes are dominated by Nostocales. Average relative abundance of the 15 more abundant bacterial orders in metagenomes from Queretaro and San Luis Potosi grouped by extraction methodology. Culture independent metagenomes are dominated by Nostocales while bacterial diversity in co-culture metagenomes is more evenly distributed.

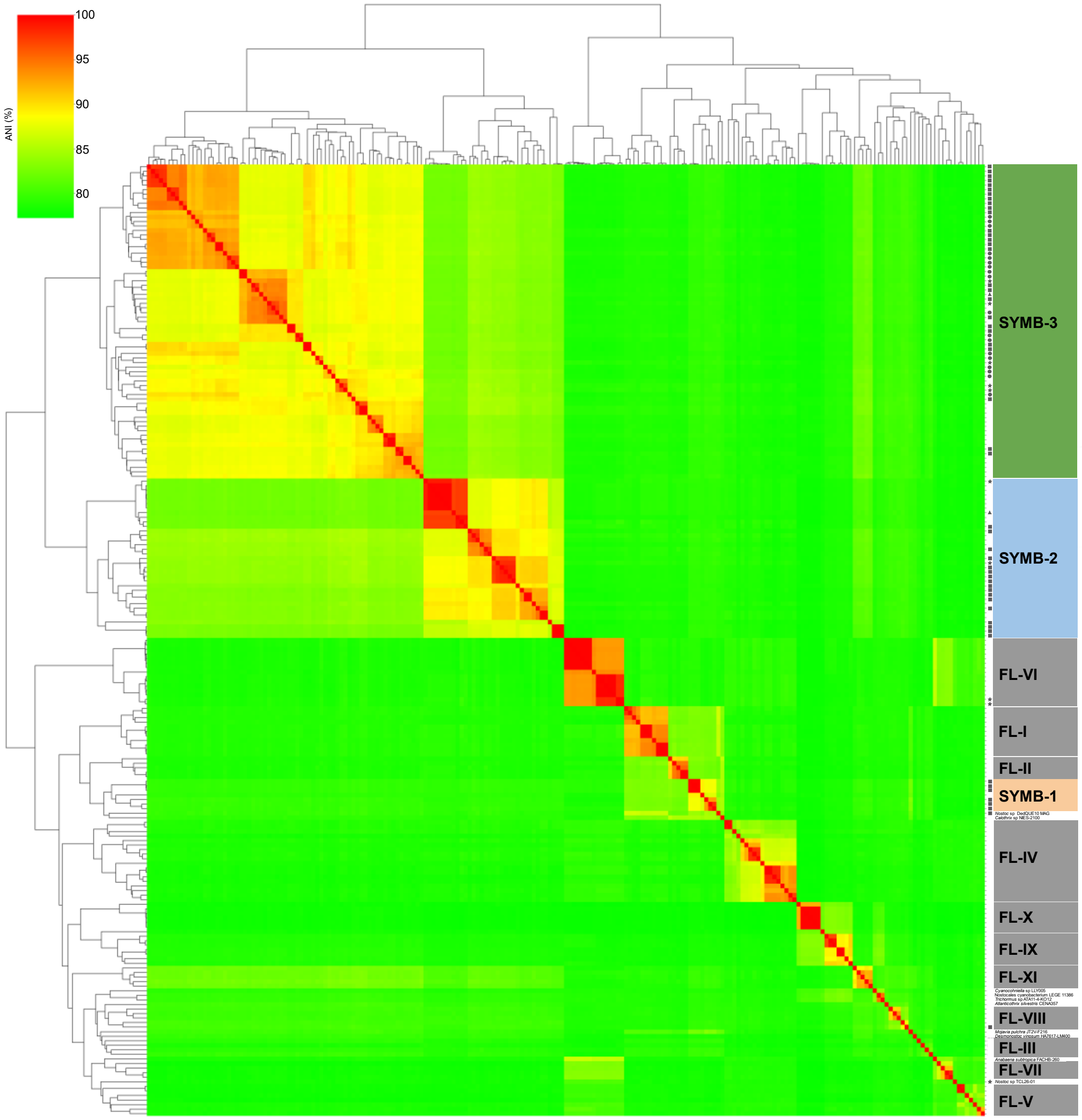

Supplementary Figure 3. ANI similarity supports cladistic grouping. ANI similarity clustermap for all 209 Nostocales genomes used for pangenomic analysis. All genomes are grouped by clade and those annotated as symbiotic are marked with the same nomenclature used in figure 3B and 3C.

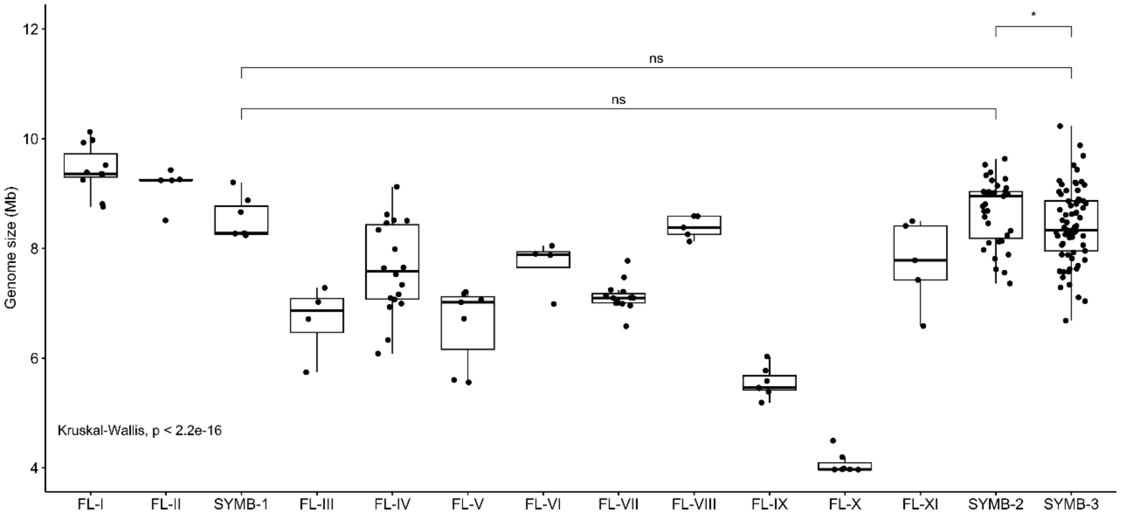

Supplementary Figure 4. All genomes in the symbiotic clades have similar sizes. The genome size of all 209 genomes used for pangenomic analysis revealed that while genome size in FL clades vary widely, it is mostly conserved between SYMB clades.

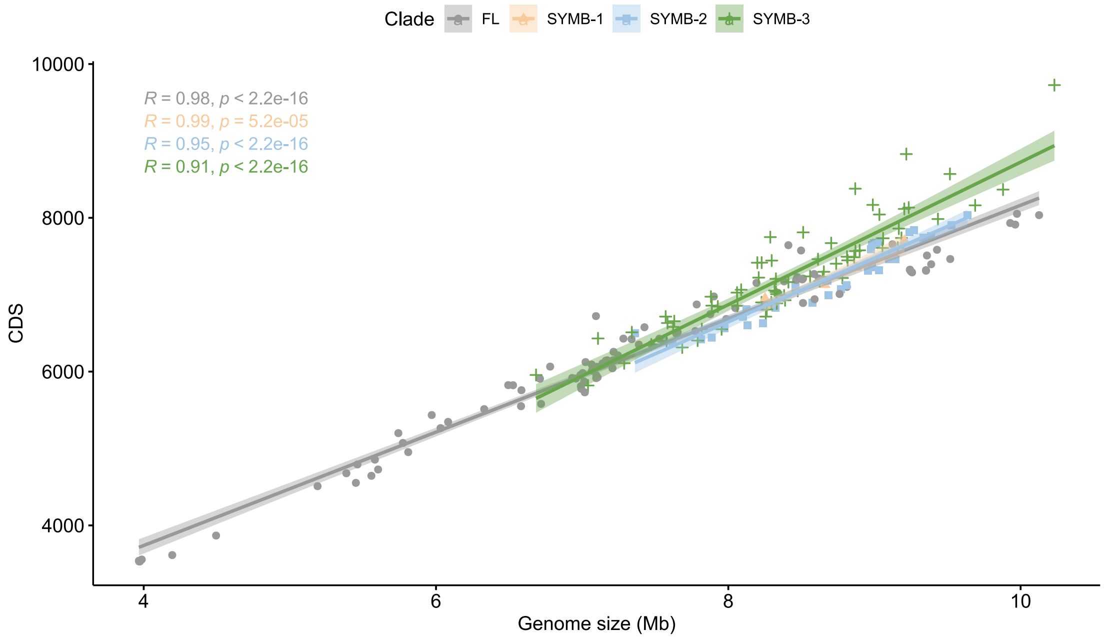

Supplementary Figure 5. All genomes have a linear relationship between size and gene content. All 209 Nostocales genomes used for pangenomic analysis have a linear correlation between size and gene content, expresses as coding sequences (CDS).

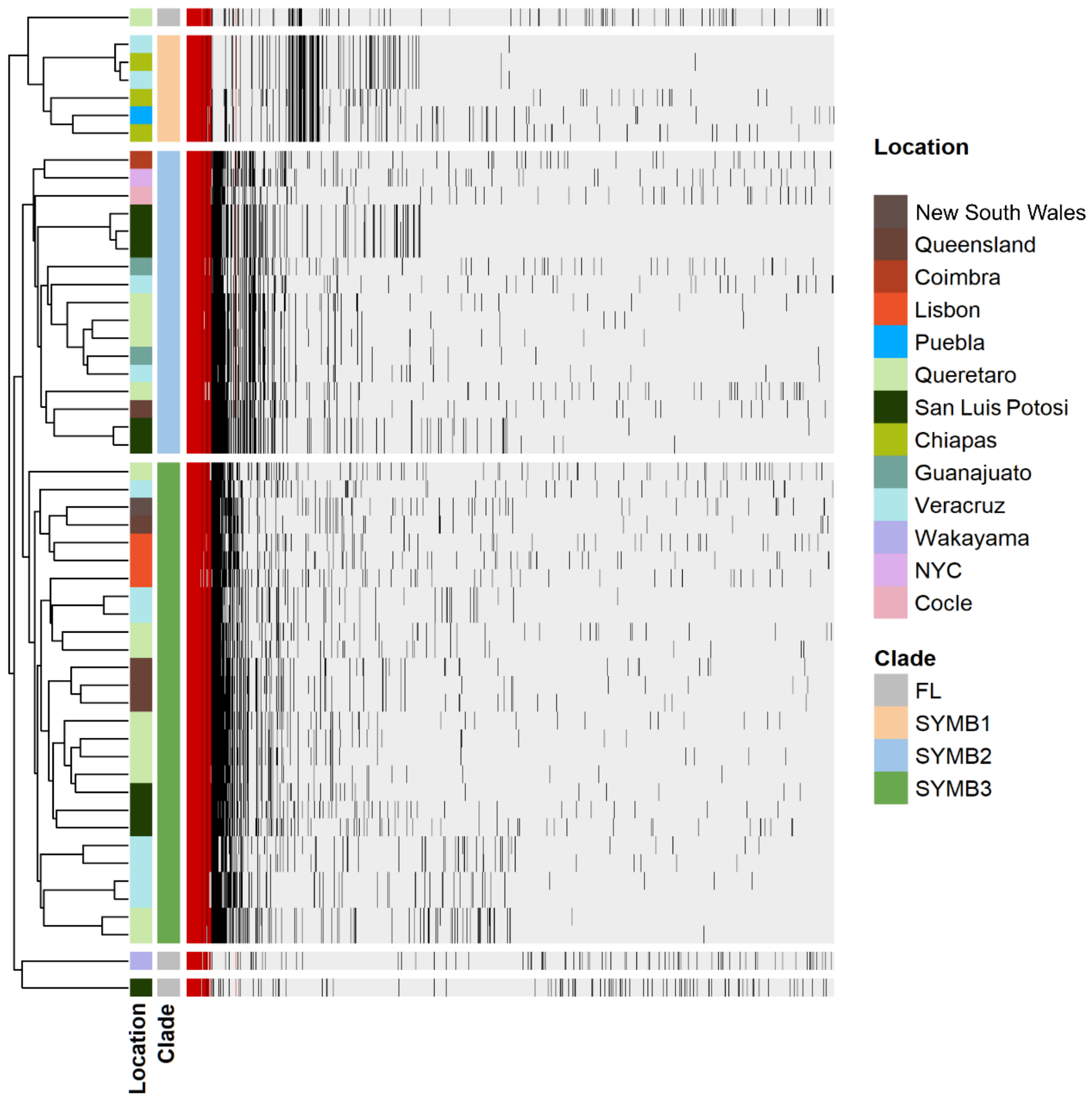

Supplementary Figure 6. Cycad cyanobiont genomes are grouped by clade. The pangenome of all cycad's cyanobiont revealed that these are grouped by clade rather than geography. Cyanobionts outside of the SYMB clades do not share homologous genes from the shell and cloud partitions (black lines) conserved in all the genomes from the SYMB clades.
